# Supplementary material for: The Construction and Exploration of a Comprehensive MicroRNA Centered Regulatory Network in Foxtail Millet (Setaria italica L.)
Source: Front Plant Sci. 2022 May 6;13:848474. doi: 10.3389/fpls.2022.848474 (PMC9121102; doi:10.3389/fpls.2022.848474)
Supplement: Supplementary file 4 [file Data_Sheet_4.zip › Sit-miR394_Seita.1G143600.1_40_TPlot.pdf]

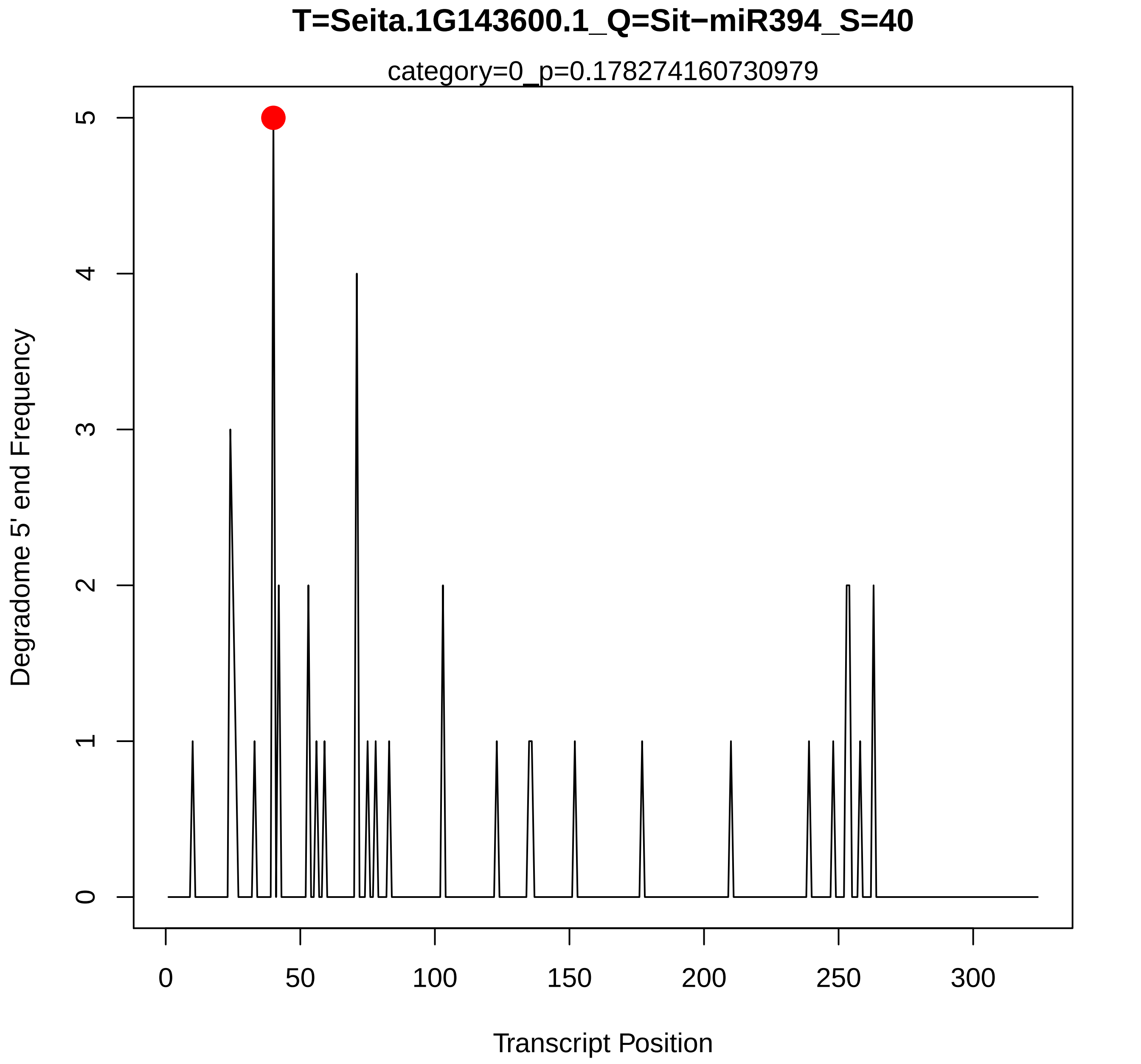
T=Seita.1G143600.1_Q=Sit−miR394_S=40
category=0_p=0.178274160730979
Degradome 5' end Frequency
0
1
2
3
4
5
0
50
100
150
200
250
300
Transcript Position
